# Epidemiology of Lateral Patellar Dislocation Including Bone Bruise Incidence: Five Years of Data from a Trauma Center

**DOI:** 10.1111/os.13933

**Published:** 2024-01-12

**Authors:** Ruilan Dai, Yue Wu, Yanfang Jiang, Hongshi Huang, Qingyang Meng, Weili Shi, Shuang Ren, Yingfang Ao

**Affiliations:** ^1^ College of Exercise and health Sciences, Tianjin University of Sport Tianjin China; ^2^ Department of Sports Medicine Peking University Third Hospital, Institute of Sports Medicine of Peking University Beijing China; ^3^ Beijing Key Laboratory of Sports Injuries Beijing China; ^4^ Engineering Research Center of Sports Trauma Treatment Technology and Devices, Ministry of Education Beijing China

**Keywords:** Bone Bruise, Distribution, Epidemiology, Incidence, Patellar Dislocation

## Abstract

**Objective:**

Systematic summary of the epidemiology of patellar dislocation is rare. This study aims to investigate sex‐, age‐, type‐, injury causing events‐, incidence of bone bruise and time from last injury (TFLI)‐specific characteristics, and detail the epidemiological characteristics of patellar dislocation.

**Method:**

In this descriptive epidemiological study, a total of 743 patients who have a history of lateral patellar dislocation with either first‐time patellar dislocation (FPD) or recurrent patellar dislocation (RPD) between August 2017 and June 2022 at our institution met the inclusion criteria and were selected in this study. Patient characteristics including the type, gender, age, events leading to patellar dislocation, incidence of patellar bone bruise, and the time from last injury (TFLI) of patellar dislocation were retrospectively obtained and described. Magnetic resonance imaging scans (MRI) of the knee were reviewed for insuring bone bruise.

**Results:**

Among the 743 patients with patellar dislocation who required surgical reconstruction of the medial retinaculum, 418 (56.2%) had RPD and 325 (43.8%) had FPD. There were more females (65.0%) than males (35.0%) in patellar dislocation patients. Among the female patients, those aged <18 years had higher incidence (31.4%) of patellar dislocation. Among the male patients, those aged <18 and 19–28 years had higher incidence (16.8%) of patellar dislocation. Of all age groups, the prevalence rate of patellar dislocation was high in juvenile population and females, but with no statistical significance. The most common patellar dislocation‐causing event was sport accidents (40.1%), followed by life accidents (23.2%). The incidence of left‐knee patellar dislocation was slightly higher than that of right‐knee patellar dislocation. The incidence of patellar bone bruise of RPD (63.2%) was significantly lower (*p* < 0.05) than that of FPD (82.2%). Patellar dislocation patients with bone bruise had shorter time from last injury (TFLI) than those without patellar bone bruise (*p* < 0.05).

**Conclusions:**

The incidence of bone bruise of RPD was lower than that of FPD, and patients with patellar bone bruise may have a shorter time from last injury than those without bone bruise.

## Graphical abstract

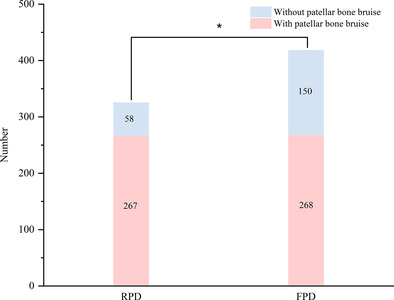

## Introduction

Patellar dislocation refers to a common clinical situation in which the patella slips to the outside of the knee joint due to various factors, such as biomechanical and anatomical abnormalities.^1^, ^2^, ^3^, ^4^, ^5^ Patellar dislocation may occur in individuals of all ages, the incidence of acute patellar dislocation has been estimated to be 43 per 100,000 children, but it is higher among adolescents, which has an incidence of 29 to 43 per 100,000 per year, and especially in females.^6^, ^7^, ^8^, ^9^ The general anatomical factors of patellar dislocation include abnormal tibial tuberosity–trochlear groove distance (TT–TG), abnormal femoral trochlear development, abnormal Q angle, patella alta, and patellar tilt, etc.^10^, ^11^, ^12^, ^13^, ^14^, ^15^, ^16^, ^17^

Although patellar dislocation is mainly a noncontact injury, most of the time, the medial patellar facet hits against the lateral femoral condyle, resulting in bone bruise between the patella and femur during the dislocation process. When complete laterally patellar displacement from the femoral trochlear groove occurs suddenly after trauma or torsion stress on the extensor mechanism, the acute external force will result in patellar restraint and concomitant injuries of medial patellar and lateral femur bone bruise, which can exist for almost a year, or even longer, before it completely disappears.^18^ The characteristics of medial patellar and lateral femur bone bruise may represent a footprint of the impact at the time of injury and provide insight into the underlying mechanisms of the pathogenesis of patellar dislocation.^19^, ^20^, ^21^ In general, after first‐time patellar dislocation (FPD), if no surgical correction is performed, it can easily occur again, eventually causing recurrent patellar dislocation (RPD).^6^, ^7^, ^22^, ^23^ Magnetic resonance imaging (MRI) is usually performed to help confirm the diagnosis of patellar dislocation, in addition to a thorough clinical examination of the knee joint, in which the bone bruise can be also observed.^4^, ^18^, ^19^, ^24^, ^25^

At present, the epidemiological studies of patellar dislocation mainly focused on a single aspect such as the cumulative annual incidence of age and sex,^26^, ^27^ and didn’t not consider such as the position of the patellar dislocation, injury events, incidence of bone bruise, and time to last injury (TFLI), so they didn’t describe the overall epidemiological characteristics of patellar dislocation. Therefore, it is necessary to conduct relevant epidemiological investigations to provide effective targeted preventive measures.

Therefore, the purpose of this study was designed to analyze the overall epidemiological characteristics of patellar dislocation. We conducted a retrospective study on the clinical data and MRI images of 854 patients with lateral patellar dislocation, 743 of whom met the inclusion criteria. This study aimed to clarify: (i) the characteristics and distribution of age, gender, laterality, injury‐causing events, incidence of patellar bone bruise, and TFLI of patellar dislocation, (ii) differences between RPD and FPD, (iii) the relationship between patellar bone bruise and the TFLI of patellar dislocation.

## Methods

### Patient Inclusion and Exclusion Criteria

Patients with patellar dislocation receiving surgical reconstruction in our institution from September 2017 to March 2022 were reviewed. Inclusion criteria were as follows: (i) the patient has a history of lateral patellar dislocation, (ii) MRI and CT scans available preoperatively. Exclusion criteria: (i) other types of patellar dislocation except lateral patellar dislocation; (ii) incomplete MRI or clinical data.

### Patellar Dislocation Data

We retrieved the medical records of the enrolled patients. The basic information of patients, such as gender, age, and demographic characteristics were recorded. MRI for the patients with patellar dislocation were collected with the use of the picture archiving and communication systems. The MRI scans were reviewed by three orthopaedic surgeons, each with more than 5 years of experience. If there was any disagreement in the diagnosis of patellar dislocation, a final decision would be made through discussion, with consensus achieved by at least two surgeons.

### Indicator Measures

Two groups, including the PPD group and RPD group, were described in this study. FPD and RPD refers to the first experience with patellar dislocation or the recurrence of patellar dislocation one or more times after conservative or surgical treatment when a patient had experienced patellar dislocation once, respectively. Preoperative MRI was performed for helping confirm the patellar dislocation. The indicator measures included the injury side, sex, injury‐causing events, the incidence of patellar bone bruise, and the time from last injury (TFLI) of patellar dislocation.

Distribution of RPD and FPD for all patients in males and females, respectively, were analyzed in this study.

Events leading to dislocation was categorized as sports accidents, life accidents, traffic accidents, unprovoked, and others not specified.

The age distribution of RPD and FPD was analyzed and divided into six age groups: 0–18 years, 19–28 years, 29–38 years, 39–48 years, 49–58 years, and 59–68 years.

Bone bruise is commonly identified on MRI following acute patellar dislocation. Since impingement occurs between the patella and femur during patellar dislocation, this study recorded only the cases when patellar and femoral bone bruise were observed at the same time. The distribution of patellar bone bruise was compared between RPD and FPD. The TFLI were compared between patients with or without patellar bone bruise.

Institutional review board approval (IRB00006761‐M2023142) was obtained before proceeding with our study, and the requirement for obtaining informed consent from the patients was waived.

### Statistical Analysis

All statistical analyses were performed using SPSS 25.0 (IBM, Chicago, IL, USA). The Kolmogorov–Smirnov test was used to evaluate whether the data conformed to a normal distribution. Descriptive data are expressed as the number, whereas non‐normal distribution data are expressed as the mean with interquartile range, M (Q1, Q3). The chi‐square test was used to analyze the distribution of male and female in different types of patellar dislocation groups. Fisher's exact test was used to analyze the age, injury side, and injury‐causing events in two groups. The Mann–Whitney U test was used to analyze the incidence of patellar bone bruise of RPD and FPD. Friedman's test was used to analyze the TFLI with or without patellar bone bruise in patients with patellar dislocation. A *p* value of <0.05 was considered to indicate statistically significant differences.

## Results

### Demographic Information

There were 854 patients with lateral patellar dislocation, 111 of whom had no MRI results, leaving a total of 743 patients who were analyzed in this study. Of the included patients, 56% (418/743) had RPD and 44% (325/743) had FPD.

### Distribution of Patellar Dislocation Type by Gender

Among the 743 patients with patellar dislocation, 483 (65.0%) were female, and 260 (35.0%) were male (Table 1). Although more females than males experience patellar dislocation, females and males had almost similar distributions between FPD and RPD.

**Table 1:** Classification of gender and injury side distribution of patellar dislocation patients.

Injury type	Male, N (percentage)			Female, N (percentage)		
	Left	Right	Both	Left	Right	Both
RPD	69 (16.50%)	69 (16.50%)	2 (4%)	156 (37.32%)	121 (28.94%)	1 (2%)
FPD	72 (22.15%)	48 (14.80%)	0	109 (33.54%)	93 (28.62%)	3 (9%)
Sum	141 (19.00%)	117 (15.75%)	2 (2%)	265 (35.67%)	214 (28.80%)	4 (5%)

Abbreviations: FPD, first‐time patellar dislocation; RPD, recurrent patellar dislocation.

### Distribution of Patellar Dislocation Type by Injury Side

Patellar dislocation may occur in the left leg, right leg, or even in both legs (Table 1). In our study, the number of patients with left patellar dislocation was higher than that with right patellar dislocation, and a few suffered bilateral patellar dislocation (left: 53.7%, right: 44.5%, bilateral: 0.8%).

### Distribution of Patellar Dislocation Type by Age

Compared with the patients with RPD, the patients with FPD had a higher proportion of those in the <18 years and 19–28 years age groups. (Table 2). The most‐affected age group was <18 years, accounting for 51.1%, followed by the age groups of 19–28 years (39.9%) and 29–38 years (13.9%). There were more female patients with patellar dislocation (*n* = 483, 65.0%) than male patients (*n* = 260, 35.0%).

**Table 2:** The distribution of RPD and FPD in different age groups by gender.

Injury type	<18 years, N (percentage)		19–28 years, N (percentage)		29–38 years, N (percentage)		39–48 years, N (percentage)		>49 years, N (percentage)	
	Male	Female	Male	Female	Male	Female	Male	Female	Male	Female
RPD	59 (7.9%)	126 (17.0%)	56 (7.5%)	106 (14.3%)	21 (2.8%)	35 (4.7%)	4 (0.5%)	8 (1.1%)	3 (0.4%)	0
FPD	59 (7.9%)	107 (14.4%)	39 (5.2%)	59 (7.9%)	18 (2.4%)	30 (4.0%)	3 (0.4%)	7 (0.9%)	1 (0.1%)	2 (0.2%)

*Note*: FPD, first‐time patellar dislocation; RPD, recurrent patellar dislocation.

### Distribution of Patellar Dislocation Type by Dislocation‐Causing Events

**FIGURE 1:**
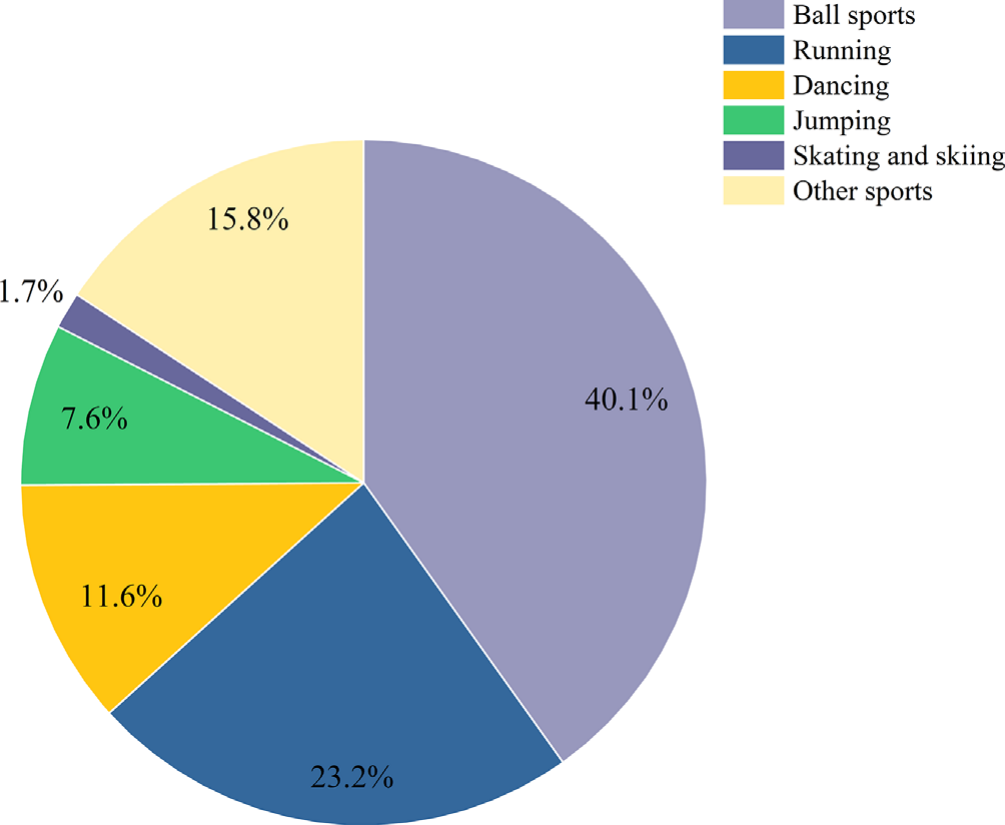
Dislocation‐causing sports. Among sports accidents, ball games with a high intensity, especially basketball, football, and table tennis, accounted for the most causes for patellar dislocation injury, followed by running and dancing sports.

No significant difference was observed between the dislocation‐causing events for the two types of patellar dislocation. The most common dislocation‐causing event was sports, including ball sports, running, dancing, and jumping, accounting for 51.4% and 57.5% in RPD and FPD, respectively, followed by life accidents, which accounted for 37.7% and 41.0%, respectively (Table 3, Figure 1).

**Table 3:** Causing events of the two types of patellar dislocation.

Injury type	Sports accidents, N (percentage)	Life accidents, N (percentage)	Traffic accidents, N (percentage)	No incentive, N (percentage)	Other courses, N (percentage)
RPD	180 (51.4%)	132 (37.7%)	8 (2.3%)	28 (8.0%)	2 (0.6%)
FPD	226 (57.5%)	161 (41.0%)	1 (0.3%)	5 (1.2%)	0 (0%)
Sum	406 (54.6%)	293 (39.4%)	9 (1.2%)	33 (4.4%)	2 (0.3%)

*Note*: *p*, FPD, first‐time patellar dislocation; RPD, recurrent patellar dislocation.

### Distribution of Patellar Dislocation Type by Incidence of Bone Bruise

**FIGURE 2:**
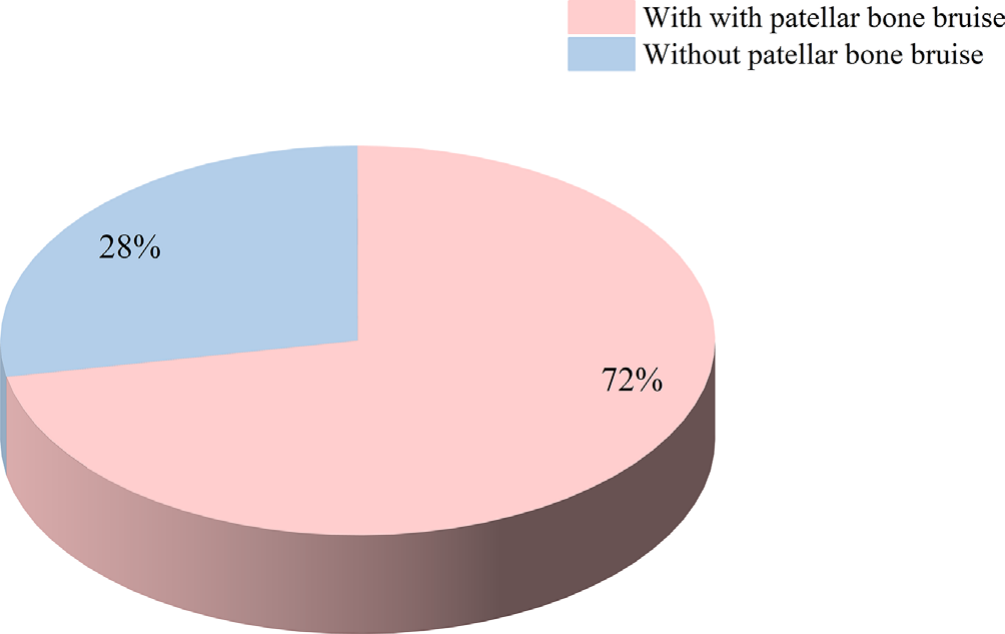
The proportion of patellar dislocation with and without patellar bone bruise.

**FIGURE 3:**
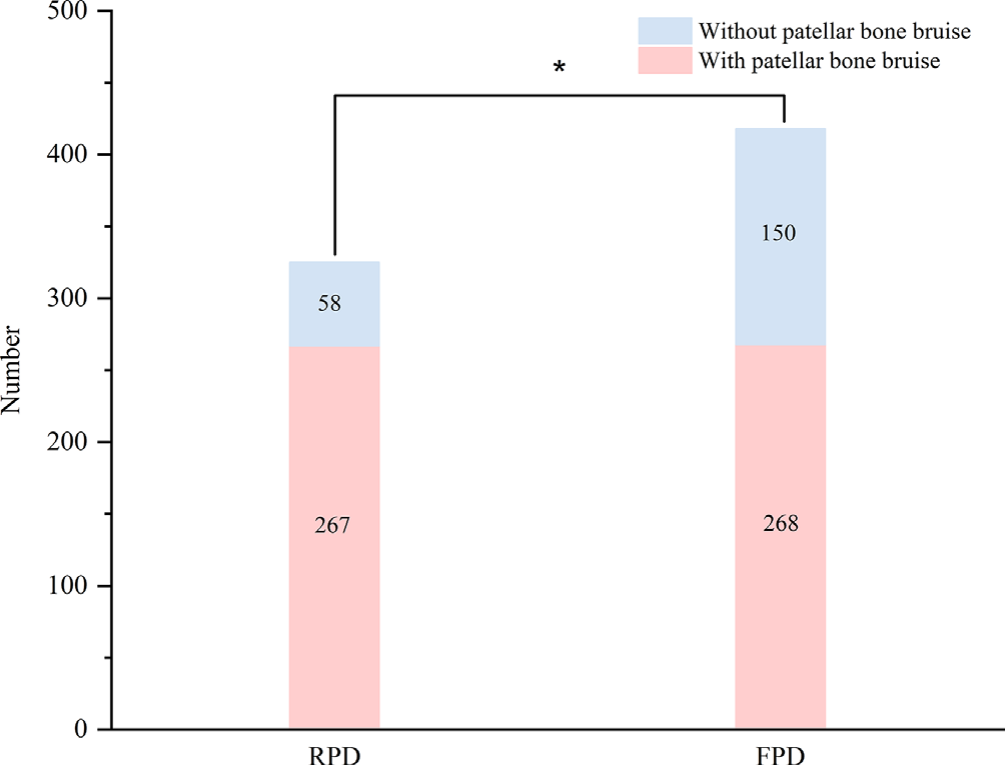
Distribution of patellar dislocation with and without patellar bone bruise for RPD and FPD. FPD had a higher incidence of patellar bone bruise than RPD (*p* = 0.001).

The incidence of patellar bone bruise was 72% in the patients with patellar dislocation (Figure 2). The proportion of patellar bone bruise in the patients with RPD (63.2%) was significantly lower than that in the patients with FPD (82.2%, *p* < 0.001) (Figure 3).

### Time from the Last Injury for the Two Types of Patellar Dislocation

**FIGURE 4:**
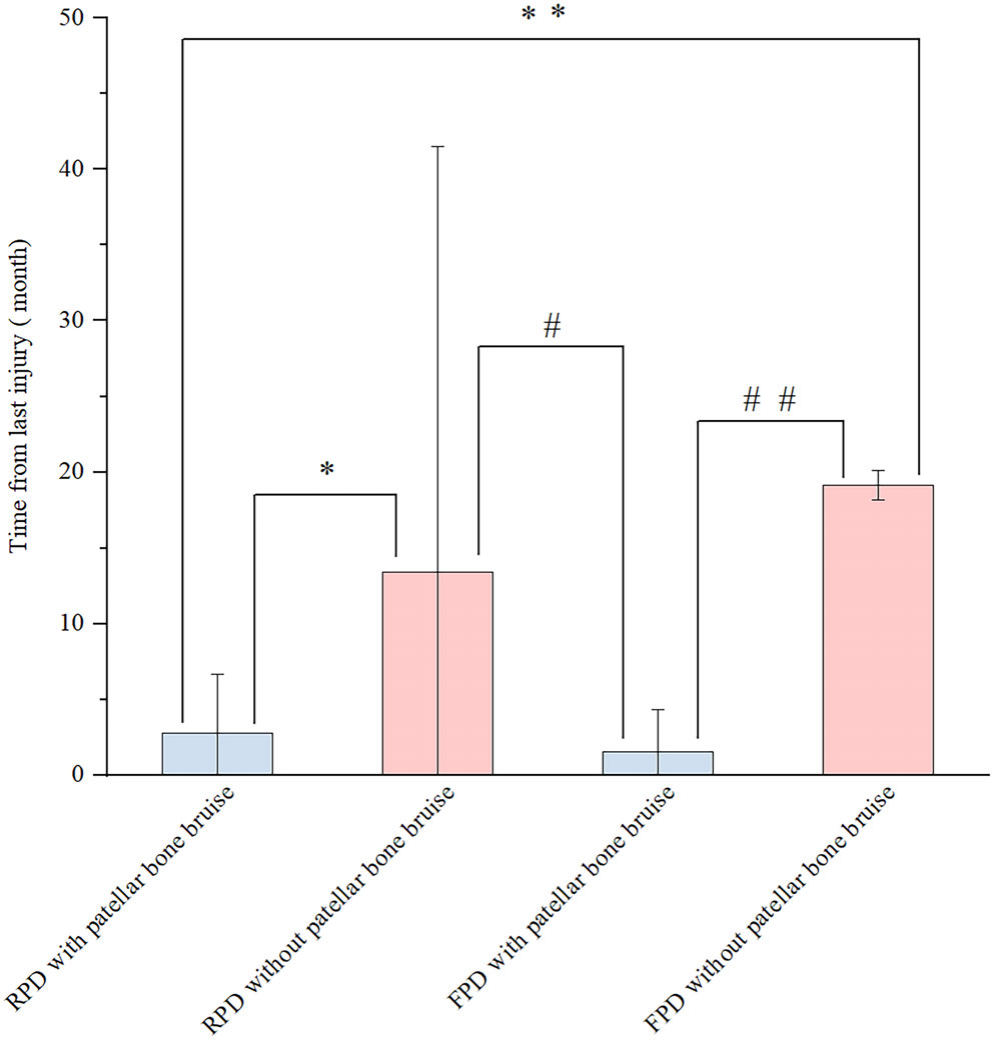
The TFLI for the two types of patellar dislocation with or without patellar bone bruise. TFLI of patellar dislocation with bone bruise was significantly shorter than that without patellar bone bruise between RPD and FPD.

The mean interval from last injury was different between FPD and RPD. TFLI of RPD with patellar bone bruise was significantly shorter (1.10 months vs. 0.53 months) than that of FPD with patellar bone bruise, and the TFLI of patellar dislocation with bone bruise was shorter (RPD: 1.10 months vs. 6 months, FPD: 0.53 months vs. 6 months, RPD vs. FPD:1.1 months vs. 6 months, *p* < 0.05) than that without patellar bone bruise. (Table 4 and Figure 4).

**Table 4:** Time from last injury in two categories of patellar dislocation with or without bone bruise.

	RPD with bone bruise	RPD without bone bruise	FPD with bone bruise	FPD without bone bruise
TFLI (m)	1.10 (0.55,3.00)	6.00 (2.00,12.60)	0.53 (0.23,2.00)	6.00 (1.00,12.00)
T	−4.926	6.005	−3.488	−4.567
P Value	0.000*, **	0.000*, ##	0.003#, ##	0.000**, ##

*Note*: RPD with bone bruise and RPD without bone bruise *, FPD with bone bruise and FPD without bone bruise # #, RPD with bone bruise and FPD without bone bruise **, FPD with bone bruise and RPD without bone bruise #, m: month, TFLI, time from last injury.

## Discussion

The goal of this study was designed to analyze the overall epidemiological characteristics of patellar dislocation. Seven hundred and forty‐three lateral patellar dislocation patients with 56% (418/743) RPD and 44% (325/743) FPD were counted and analyzed in this study.

### The Incidence of Bone Bruise Was Different in RPD and FPD.

The most important finding of this study was that the overall incidence of patellar dislocation with patellar bone bruise in this study was different than the previous cohorts,^19^, ^28^ and it was markedly different between FPD and RPD.

The incidence of patellar bone bruise in RPD was 63.2%, whereas it was 85% in FPD, which was in contrast with the previous study where the incidence of bone bruise of the lateral femoral condyle and medial patella border were up to nearly 100% of patients after primary patellar dislocation.^26^, ^29^ The lower incidence of patellar dislocation with patellar bone bruise observed in our study may reflect an overall dislocation category in the general patellar dislocation population, which has been shown to affect the lateral patellofemoral retinaculum laxity after FPD,^30^, ^31^ so the incidence of patellar bone bruise RPD was lower than that of FPD. Furthermore, the distribution of bone bruise may represent the relative motion tracking, like a footprint left during injury to the patella and femur in the process of patellar dislocation, which could provide a basis for understanding the pathogenic mechanism of patellar dislocation.^21^, ^32^, ^33^ Bone bruise in patellar dislocation may be related to age and anatomical characteristics since Zhang et al. have reported that there was a difference in the location of MPFL injury between children and adolescents, MPFL is most easily injured at its patellar insertion in children.^33^ However, Tompkins et al. have reported that underlying anatomic patellar dislocation risk factors do not predict injury patterns as no clear relationship was found between the severity and location of bone bruise.^32^ For children and adolescents, abundant physical strength and intense exercise are more likely to lead to patellar dislocation injury and bone bruise in patellar instability patients although there was no significant difference.^19^

### The Relationship between Bone Bruise and the Time from the Last Injury of Patellar Dislocation.

The second important finding of this study was that, regardless of whether it was FPD or RPD, depending on whether they had patellar bone bruise, the TFLI of patellar dislocation without patellar bone bruise was significantly longer than that with patellar bone bruise. This would be related to the process that bone bruise signal alteration tends to disappear gradually.^34^ The TFLI of patellar dislocation without patellar bone bruise was significantly longer than that with patellar bone bruise in this study, this was probably due to that the sample size of the previous study being relatively small, and no comparison was conducted between FPD and RPD. Since after suffering the FPD, the medial patellar retinaculum will be damaged and become looser, which can make it easy for patellar dislocation to happen again. If no surgery was performed, the frequency of patella dislocation will increase, even if there was no bone bruise when it occurs again. This suggests that if a patient who describes himself as FPD does not have bone bruise, he is probably not FPD but RPD, or he has a history of patellar dislocation, but he cannot remember it.

### The Characteristics and Distribution of Patellar Dislocation

According to our study, the ratio of males and females with patellar dislocation varies, 64.5% females and 35.5% males were recorded in this study, which was consistent with previous studies, that females are more prone in patellar dislocation.^6^, ^35^ Among those patients, 56% of them were suffering RPD while 44% were suffering FPD. Previous studies have reported that after RPD, it may occur again in more than 50% of patients with FPD.^36^ As anatomical risk factors of patellar instability include morphological of patella or femur condyle with abnormal shape as well as depth of the trochlear groove,^37^ an abnormal femoral condyle shape would be low‐grade (type A) or high‐grade trochlear dysplasia (types B‐D),^38^ and the depth of the femoral trochlea become shallower and the groove angle showed an increase with age in females, which was also the reason why females had more patellar instability than males before adolescence.^35^, ^39^ We conducted statistics on the activities of patients with patellar dislocation, and found that more than half of the patients suffering patellar dislocation partly because of sports accidents, followed by life accidents such as squatting or going down stairs. Higher intensity exercise, especially basketball, football, and table tennis, account for the most causes of patellar dislocation as reported by Höhne et al.^40^

In terms of age group, the majority of patients with patellar dislocation were adolescents, followed by young adults, this was consistent with the results of the study by Hasler.^41^ The osseous sulcus angle, cartilaginous sulcus angle, and patella sulcus angle decreased (became deeper) with age until after 8 years and then plateaued, so children are more likely to suffer from patellofemoral instability than other age groups.^42^, ^43^

Few articles reported on which leg of patients with patellar dislocation tended to occur. The authors found that the majority of patients with patellar dislocation were left leg dislocations, which was the same for PFD and RPD. Kinematic differences that exist between the dominant and nondominant legs may elucidate the mechanisms and risk of sports injury may also be associated with leg dominance.

### Strengths and Limitations

The study conducted a relatively large sample size of patellar dislocation and found that the incidence of bone bruise in patients with patellar dislocation was different and was significantly lower than previously studies have reported, and the incidence of bone bruise in RPD was significantly lower than those in FPD. In addition, we also found that patients with patellar dislocation with bone bruise had a shorter injury TFLI and patients without patellar bone bruise had a longer injury TFLI, which together with the above results can assist clinicians to determine whether the patient is FPD or RPD.

There are several limitations of this study that should be noted. First, because it was a retrospective study, bias was inevitable. Second, the cases were from only a single center, and other factors, such as mechanism of injury, occupation, and classification of types of anatomical abnormalities, were not analyzed. Therefore, a multicenter study that analyzes additional related factors in an expanded sample size should be performed in the future.

## Conclusion

Epidemiology of the patient cohorts, including the incidence of bone bruise, and time from last injury (TFLI) were significantly different between first‐time patellar dislocation (FPD) and recurrent patellar dislocation (RPD). Sport accidents was more likely to cause lateral patellar dislocation, and females had higher proportions of patellar dislocation than males though there was no statistical significance. RPD had lower incidence of bone bruise than FPD, and patients with patellar bone bruise may have a shorter time from last injury than those without bone bruise.

## Conflict of Interest Statement

Each author certifies that he or she has no commercial associations (e.g., consultancies, stock ownership equity interest, paten/licensing arrangements, etc.) that might pose conflict of interest in connection with the submitted article.

## Ethical Statement

Ethical approval (IRB00006761‐M2023142) was obtained from the universities ethics committee and written informed consent was attained from all participants.

## Author Contributions

Yingfang Ao conceived the idea for the study and then reviewed and edited the manuscript, Ruilan Dai and Shuang Ren designed the study, Ruilan Dai, Shuang Ren, Yue Wu, Yanfang Jiang, Hongshi Huang, Qingyang Meng, and Weili Shi collected the relevant data. Ruilan Dai drafted the manuscript. Ruilan Dai, Yue Wu, and Shuang Ren performed the statistical analyses. All authors reviewed and revised the manuscript critically, read and approved the final manuscript.
